# Supplementary material for: Measurement Methods Used to Assess the School Food Environment: A Systematic Review
Source: Int J Environ Res Public Health. 2020 Mar 3;17(5):1623. doi: 10.3390/ijerph17051623 (PMC7084932; doi:10.3390/ijerph17051623)
Supplement: Supplementary file 1 [file ijerph-17-01623-s001.pdf]

Supplementary material Figure S1: Criteria and standards for quality assessment of the school food environment measurement methods

Criteria for assessment	Standards
Comprehensiveness	Refers to the ability of the methods and tools to adequately capture key aspects of food provision.<br><br>The proportion of the four environmental dimensions ((physical, economic, policy and sociocultural) as defined by Swinburn et. al. (1) to categorise the methods used to measure the school food environment.<br><br>'Low' comprehensiveness means only 1 dimension was included.<br><br>'Medium' comprehensiveness means 2-3 dimension of the framework were included.<br><br>'High' comprehensiveness means 4 dimension of the framework were included.
Generalisability	Refers to the degree to which the results of the study are generalizable to other groups of people or contexts. The methods and tools used in the studies can be assessed as follows:<br><br>'Low' if the methods and tools are mostly country-specific.<br><br>'Medium' if they are applicable in other countries/contexts.<br><br>'High' if they are applicable globally.
Relevance	Refers to the degree to which the methods of the study are relevant and accurate to the people and situations assessed by the studies. The criterion takes into account the following aspects: the food environment is assessed by one or more than one measurement method, measurement tools are pre-tested; the sample is representative. The process for assessing relevance can be described in detail by the following<br><br>'Low' if the study meets 1 of 3sub-criteria or does not meet any sub-criteria<br><br>'Medium' if a study meets 2 of 3 sub-criteria<br><br>'High' if a study meets at least 3 of 3 sub-criteria
Feasibility	Refers to the ease and practicality of applying the methods and tools. The following aspects should be taken into account for the assessment: easy to administer (e.g. low human resource and technical skills required), and interpretable (being supplemented by detailed instruction or guides for using the methods and tools and interpreting results).<br><br>The feasibility was assessed based on the authors' judgement by comparing the relative feasibility across these studies. Each study was rated as 'high', 'medium' or 'low' feasibility.

**Criteria for assessment**	**Standards**
**Overall assessment**	The overall quality of the methods and tools for each study was rated as 'Low', 'Medium' or 'High' based on the collective assessment for the four individual assessment criteria as follows:<br><br>Low: Two or more Low ratings for any of the assessment criteria.<br><br>Medium: Three High plus one Low rating for any of the assessment criteria; four Medium ratings; two High plus one Medium plus one Low rating for any of the assessment criteria; one High plus three Medium ratings for any of the assessment criteria; three Medium plus one Low rating for any of the assessment criteria; or one High plus two Medium and one Low rating for any of the assessment criteria.<br><br>High: Four High ratings; three High plus one Medium rating for any of the assessment criteria; or two High plus two Medium ratings for any of the assessment criteria.<br><br>Those studies which provided insufficient information to assess against any individual criteria were rated as not applicable (N/A) for the overall quality assessment of methods and tools.
